# Enhancement of the proline and nitric oxide synthetic pathway improves fermentation ability under multiple baking-associated stress conditions in industrial baker's yeast

**DOI:** 10.1186/1475-2859-11-40

**Published:** 2012-04-01

**Authors:** Yu Sasano, Yutaka Haitani, Keisuke Hashida, Iwao Ohtsu, Jun Shima, Hiroshi Takagi

**Affiliations:** 1 Graduate School of Biological Sciences, Nara Institute of Science and Technology, 8916-5 Takayama, Ikoma, Nara 630-0192, Japan; 2 Research Division of Microbial Sciences, Kyoto University, Kitashirakawa Oiwake-cho, Sakyo-ku, Kyoto 606-8502, Japan

**Keywords:** Baker's yeast, Proline, Mpr1, Nitric oxide, Baking-associated stress tolerance

## Abstract

**Background:**

During the bread-making process, industrial baker's yeast, mostly *Saccharomyces cerevisiae*, is exposed to baking-associated stresses, such as air-drying and freeze-thaw stress. These baking-associated stresses exert severe injury to yeast cells, mainly due to the generation of reactive oxygen species (ROS), leading to cell death and reduced fermentation ability. Thus, there is a great need for a baker's yeast strain with higher tolerance to baking-associated stresses. Recently, we revealed a novel antioxidative mechanism in a laboratory yeast strain that is involved in stress-induced nitric oxide (NO) synthesis from proline via proline oxidase Put1 and *N*-acetyltransferase Mpr1. We also found that expression of the proline-feedback inhibition-less sensitive mutant γ-glutamyl kinase (Pro1-I150T) and the thermostable mutant Mpr1-F65L resulted in an enhanced fermentation ability of baker's yeast in bread dough after freeze-thaw stress and air-drying stress, respectively. However, baker's yeast strains with high fermentation ability under multiple baking-associated stresses have not yet been developed.

**Results:**

We constructed a self-cloned diploid baker's yeast strain with enhanced proline and NO synthesis by expressing Pro1-I150T and Mpr1-F65L in the presence of functional Put1. The engineered strain increased the intracellular NO level in response to air-drying stress, and the strain was tolerant not only to oxidative stress but also to both air-drying and freeze-thaw stresses probably due to the reduced intracellular ROS level. We also showed that the resultant strain retained higher leavening activity in bread dough after air-drying and freeze-thaw stress than that of the wild-type strain. On the other hand, enhanced stress tolerance and fermentation ability did not occur in the *put1*-deficient strain. This result suggests that NO is synthesized in baker's yeast from proline in response to oxidative stresses that induce ROS generation and that increased NO plays an important role in baking-associated stress tolerance.

**Conclusions:**

In this work, we clarified the importance of Put1- and Mpr1-mediated NO generation from proline to the baking-associated stress tolerance in industrial baker's yeast. We also demonstrated that baker's yeast that enhances the proline and NO synthetic pathway by expressing the Pro1-I150T and Mpr1-F65L variants showed improved fermentation ability under multiple baking-associated stress conditions. From a biotechnological perspective, the enhancement of proline and NO synthesis could be promising for breeding novel baker's yeast strains.

## Background

Baker's yeast (mostly strains of *Saccharomyces cerevisiae*) is exposed to various baking-associated stresses such as air-drying, high temperature, freeze-thaw, and high osmotic pressure during bread making [1]. Dried yeast is widely used for bread making because it has a longer storage time and lower transport costs than compressed yeast. During the preparation process for dried yeast, yeast cells are exposed to air-drying stress, which exerts many harmful influences such as the accumulation of misfolded proteins [2], mitochondrial dysfunction, and vacuolar acidification [3], leading to lowered fermentation ability. Thus, air-drying stress tolerance is a necessary characteristic of baker's yeast for dried yeast preparation. During the drying process, the flow of hot air increases the temperature of yeast cells to around 37°C. Therefore, air-drying stress is considered to be a combination of two stresses, high temperature and dehydration.

The recent development of frozen dough baking technology is valuable because it improves the labor conditions in the bakery industry and enables consumers to purchase fresh bread. However, freezing and the subsequent thawing treatments cause severe injury to yeast cells and lower the leavening ability. For this reason, the development of a baker's yeast strain that is tolerant to freezing stress is desirable. In addition, yeast cells sometimes encounter such stresses in a multiple and sequential manner [1]. Thus, the baking industry requires yeast strains with tolerance to multiple baking-associated stresses. Both air-drying and freeze-thaw stresses are reported to accumulate intracellular reactive oxygen species (ROS), such as superoxide anion, hydrogen peroxide, and hydroxyl radical [3-5]. During normal respiratory metabolism in all aerobic organisms including yeast, several ROS, which are produced as byproducts, are scavenged by a variety of antioxidant enzymes. However, the transient heat shock and loss of water can promote dysfunctions in the enzymes capable of detoxifying ROS. As a result, the increased ROS levels damage cellular components such as lipids, proteins, and nucleic acids, leading to low fermentation ability or cell death [6].

**Figure 1:**
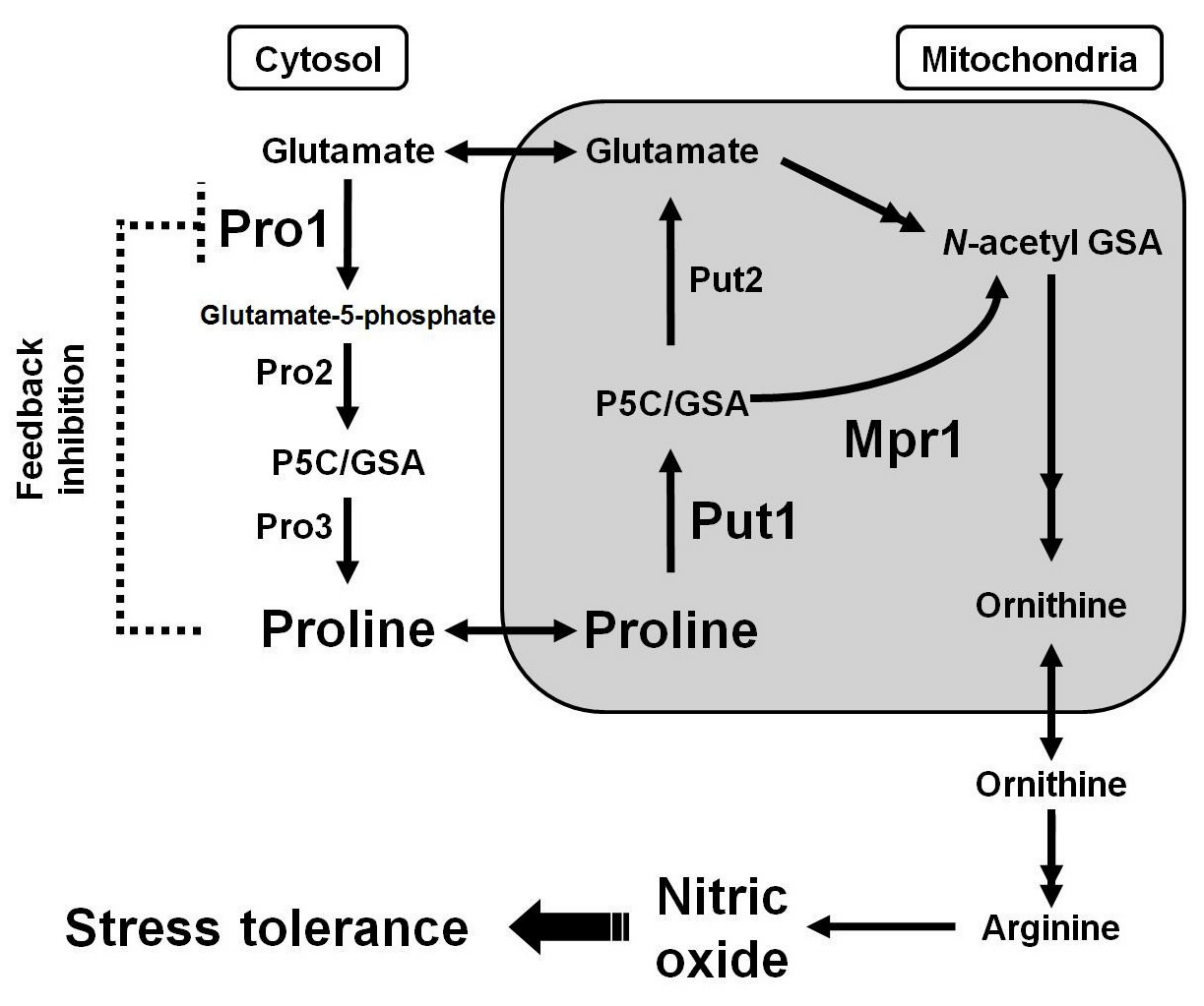
**Metabolic pathway of proline and arginine in *Saccharomyces cerevisiae***. Protein names: Pro1, γ-glutamyl kinase; Pro2, γ-glutamyl phosphate reductase; Pro3, P5C reductase; Put1, proline oxidase; Put2, P5C dehydrogenase; Mpr1; P5C/GSA *N*-acetyltransferase.

We previously found that laboratory yeast strains with proline accumulation by expressing the proline-feedback inhibition-less sensitive mutant -glutamyl kinase (Pro1-I150T) were tolerant to various stresses, including freezing, desiccation, oxidation, and ethanol (Figure 1) [7-12]. Proline has many functions *in vitro*, such as protein and membrane stabilization, lowering the *T*_m _value of DNA, and scavenging of ROS, but the mechanisms of these functions *in vivo *are poorly understood [13]. Recently, we reported that proline-accumulating baker's yeast retained higher-level fermentation ability in both frozen dough and sweet dough than that of the wild-type strain [14,15].

*N*-Acetyltransferase Mpr1 was shown to decrease the intracellular ROS levels when yeast cells are exposed to oxidative stresses such as heat-shock, hydrogen peroxide, freezing, or ethanol treatment [16-18]. Interestingly, Mpr1 acetylates the proline catabolism intermediate Δ^1^-pyrroline-5-carboxylate (P5C) and its equilibrium compound, glutamate-γ-semialdehyde (GSA), under oxidative stress conditions (Figure 1) [18]. Because *N*-acetyl GSA, the reaction product of Mpr1, is an intermediate of arginine synthesis, Mpr1 mediates the proline and arginine metabolic pathway via the acetylation of P5C or GSA. In our previous study, we isolated the Mpr1 variant with enhanced thermal stability (Mpr1-F65L) [19]. An industrial baker's yeast strain expressing Mpr1-F65L showed higher cell viability and leavening activity after air-drying stress [20].

Recently, we found that nitric oxide (NO) is produced under oxidative stress conditions from proline via proline oxidase Put1 and Mpr1 in *S. cerevisiae *Σ1278b strain (Figure 1) [21]. In mammals, NO as a signaling molecule is involved in stress tolerance through the activation of soluble guanylate cyclase and the posttranslational modification such as *S*-nitrosylation [22,23]. The Put1- and Mpr1-mediated NO generation in yeast may also confer stress tolerance by the same mechanism. Although it is unclear whether a similar mechanism exists in industrial baker's yeast, the fact that the disruption of the *MPR *gene in a baker's yeast strain resulted in air-dry stress sensitivity suggests the importance of the *MPR *gene and the existence of the same antioxidative mechanism in baker's yeast [20].

Based on these findings, we speculated that enhancement of the proline and NO synthetic pathway confers tolerance to multiple baking-associated stresses such as air-drying and freeze-thaw stress on baker's yeast. For the application of recombinant yeasts for commercial use, a self-cloning yeast that has no foreign genes or DNA sequences except for yeast DNA might be more acceptable for consumers than a genetically modified yeast. In this study, we constructed a self-cloned diploid baker's yeast strain with enhanced proline and NO synthesis by expressing Pro1-I150T and Mpr1-F65L in the presence of functional Put1. The resultant strain showed a significantly higher intracellular NO level and a lower intracellular ROS level than those in the wild-type strain, leading to tolerance to multiple baking-associated stresses. An increase in intracellular proline and NO levels also enhanced the leavening activity in bread doughs after air-drying and freeze-thaw stress treatment compared with the wild-type strain.

## Results

### Intracellular proline contents of baker's yeast strains

We have already shown that proline accumulation with the expression of Pro1-I150T confers oxidative stress tolerance and fermentation ability after freezing stress [14] and high-sugar stress [15] using the *put1*-disrupted strain. In addition, we revealed that a diploid baker's yeast strain that simultaneously expresses the Pro1-I150T and Mpr1-F65L variants showed enhanced the fermentation ability after air-dry stress [20]. However, this strain (PRO-F65L [20]) lacked the functional *PUT1 *gene required for NO synthesis from proline (Figure 1). To enhance the NO synthetic pathway from proline, we constructed a diploid baker's yeast strain with the wild-type *PUT1 *gene, which expresses the Pro1-I150T and Mpr1-F65L variants (PRO1-I150T/MPR1-F65L), in addition to PRO1-I150T/MPR1-F65L/Δput1, which is identical to PRO-F65L [20] (Table 1).

**Table 1:** *S.cerevisiae *strains used in this study

Strain	Genotype	Background and/or description
3346	*α PRO1 MPR2*	Haploid [14]

3347	*α PRO1 MPR2*	Haploid [14]

3346 PRO1-I150T/MPR1-F65L	*α pro1-I150T mpr1-F65L*	3346-ura3 [14]

3347 PRO1-I150T/MPR1-F65L	*α pro1-I150T mpr1-F65L*	3347-ura3 [14]

WT	*α*/*α*	Diploid derived from 3346 and 3347, wild-type

PRO1-I150T/MPR1-F65L	*α*/*α pro1-I150T/pro1-I150T* *mpr1-F65L/mpr1-F65L*	Diploid derived from 3346 Pro1-I150T/Mpr1-F65L and 3347 Pro1-I150T/Mpr1-F65L

PRO1-I150T/MPR1-F65L/Δput1	*α*/*α pro1-I150T/pro1-I150T* *mpr1-F65L/mpr1-F65L put1::URA3/put1::URA3*	Identical to PRO-F65L strain [20]

**Figure 2:**
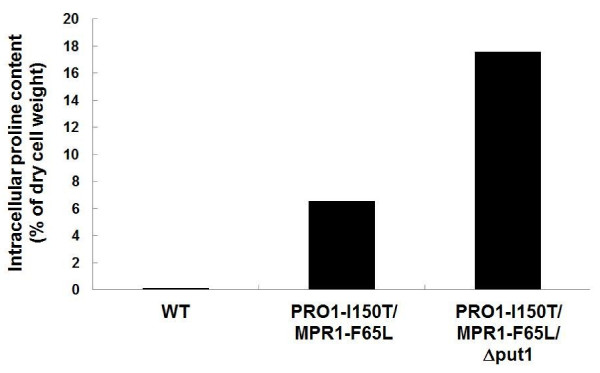
**Intracellular proline contents of baker's yeast strains**. Cells of WT, Pro1-I150T/MPR1-F65L, and Pro1-I150T/MPR1-F65L/Δput1 were precultured at 30°C for 48 h in cane molasses medium, washed twice, and then intracellular proline was extracted by the method described in Methods section. The proline content is expressed as a percentage of the dry cell weight. The data shown are from one experiment. Similar results were seen in replicates of this experiment.

To confirm proline accumulation in the cells, we measured the intracellular proline content by an amino acid analyzer (Figure 2). The amounts of intracellular proline in strains WT, PRO1-I150T/MPR1-F65L, and PRO1-I150T/MPR1-F65L/Δput1 were 0.16%, 6.54%, and 17.6%, respectively, indicating that both PRO1-I150T/MPR1-F65L and PRO1-I150T/MPR1-F65L/Δput1 accumulated proline. As expected, the PRO1-I150T/MPR1-F65L/put1 strain accumulated much more proline than did the PRO1-I150T/MPR1-F65L strain due to the lack of conversion of proline into glutamate or arginine.

### Baking-associated stress tolerance of baker's yeast strains

**Figure 3:**
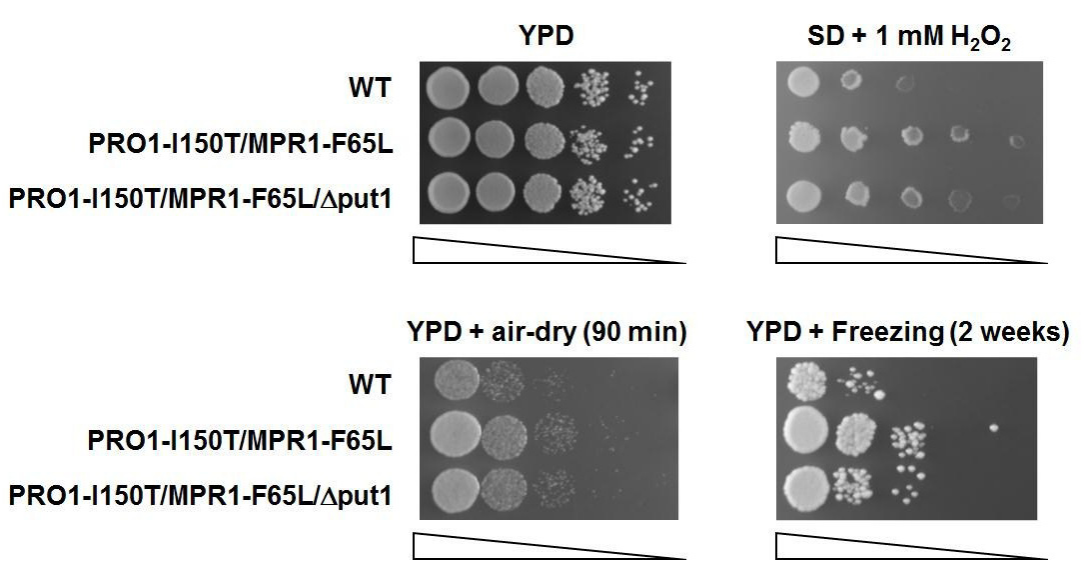
**Growth phenotypes of baker's yeast strains under baking-associated stress conditions**. Yeast cells after cultivation in cane molasses medium were harvested, washed twice, then serially diluted and spotted onto YPD medium and SD containing 1 mM hydrogen peroxide (H_2_O_2_) medium. After air-drying stress and freeze-thaw stress, yeast cells were spotted onto YPD plate. Approximately 1.5 × 10^7 ^cells of each strain and 10^-1 ^to 10^-4 ^serial dilutions (from left to right, as indicated by triangles) were spotted onto each plate except for SD + 1 mM H_2_O_2 _plate (4^-1 ^to 4^-4 ^serial dilutions). The plates were incubated at 30°C for 1 or 2 days.

Next, we tested growth phenotypes of baker's yeast strains before and after baking-associated stress treatments. As shown in Figure 3, the PRO1-I150T/MPR1-F65L strain was more resistant to the oxidative stress of hydrogen peroxide than were the other strains. When yeast cells were exposed to air-drying and freeze-thaw stresses, both PRO1-I150T/MPR1-F65L and PRO1-I150T/MPR1-F65L/Δput1 showed a greater tolerance than that of WT, indicating that intracellular proline accumulation conferred tolerance to these stresses on baker's yeast. Interestingly, PRO1-I150T/MPR1-F65L exhibited higher tolerance than that of PRO1-I150T/MPR1-F65L/Δput1 under both stress conditions (Figure 3). This result suggests that Put1 is involved in baking-associated stress tolerance in baker's yeast strains.

### Intracellular ROS levels of baker's yeast strains after air-drying stress

**Figure 4:**
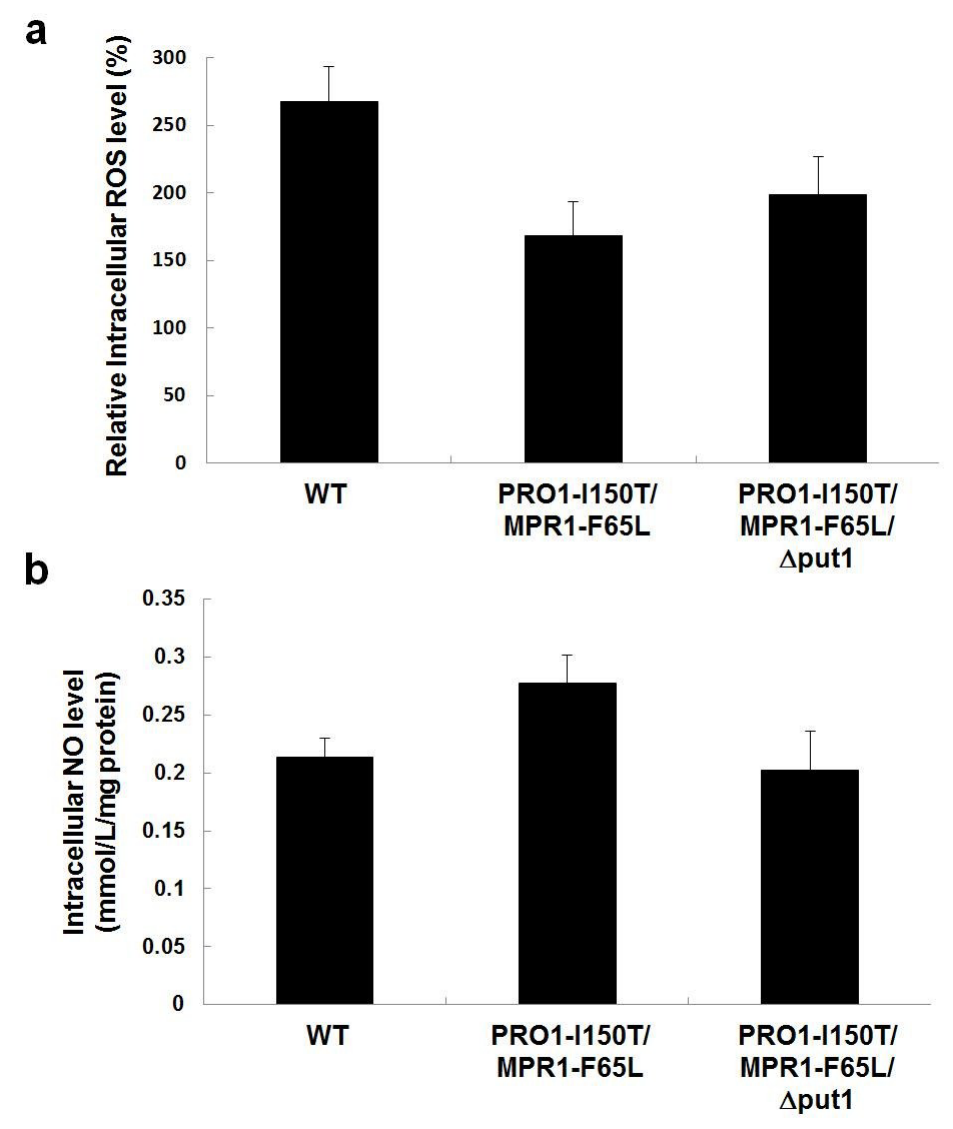
**Intracellular levels of ROS and NO of baker's yeast strains after air-drying stress treatment**. (**a**) Intracellular oxidative levels after air-drying stress was measured by the method described in Methods section. The fluorescence intensity before stress treatment of each strain was defined as 100%. The values are the means and standard deviations of results from four independent experiments. Significant difference of PRO1-I150T/MPR1-F65L from WT was confirmed by Student's *t *test (*P *< 0.005). (**b**) Intracellular NO levels after air-drying stress was measured by the method described in Methods section. The values are the means and standard deviations of results from four independent experiments. Significant difference of PRO1-I150T/MPR1-F65L from WT and PRO1-I150T/MPR1-F65L from Pro1-I150T/MPR1-F65L/Δput1 was confirmed by Student's *t *test (*P *< 0.01) and (*P *< 0.05), respectively.

The PRO1-I150T/MPR1-F65L strain showed higher tolerance to hydrogen peroxide than that of other strains (Figure 3). It was reported that severe desiccation and freezing stresses induce intracellular accumulation of ROS [3-5]. Thus, to examine why the PRO1-I150T/MPR1-F65L strain showed higher tolerance to baking-associated stresses than that of other strains, we measured the intracellular ROS levels of baker's yeast strains after air-drying stress (Figure 4a). Crude extracts from the WT strain showed a 2.5-fold increase in fluorescence after exposure to air-drying. The oxidation level was reduced by approximately 25-40% in proline-accumulating strains (PRO1-I150T/MPR1-F65L and PRO1-I150T/MPR1-F65L/Δput1). Corresponding to the data of air-drying stress tolerance (Figure 3), the intracellular ROS level of PRO1-I150T/MPR1-F65L was significantly lower than that of PRO1-I150T/MPR1-F65L/Δput1.

### Intracellular NO levels of baker's yeast strains after air-drying stress

Recently, we obtained evidence suggesting that NO is produced by an increased level of arginine and contributes to oxidative stress tolerance in yeast cells [21]. To further investigate the reason for reduced ROS levels, we measured intracellular NO levels of baker's yeast strains after air-drying stress (Figure 4b). Interestingly, the PRO1-I150T/MPR1-F65L strain showed a higher level of NO than those of the WT and PRO1-I150T/MPR1-F65L/Δput1 strains. This result suggests that enhancement of proline and arginine synthesis requiring Pro1-I150T, Put1, and Mpr1-F65L is indispensable for an increase in NO content in response to air-drying stress. The increased NO level might confer tolerance to both oxidative and baking-associated stresses on baker's yeast cells.

### Fermentation abilities of baker's yeast strains on bread dough under multiple baking-associated stress conditions

**Figure 5:**
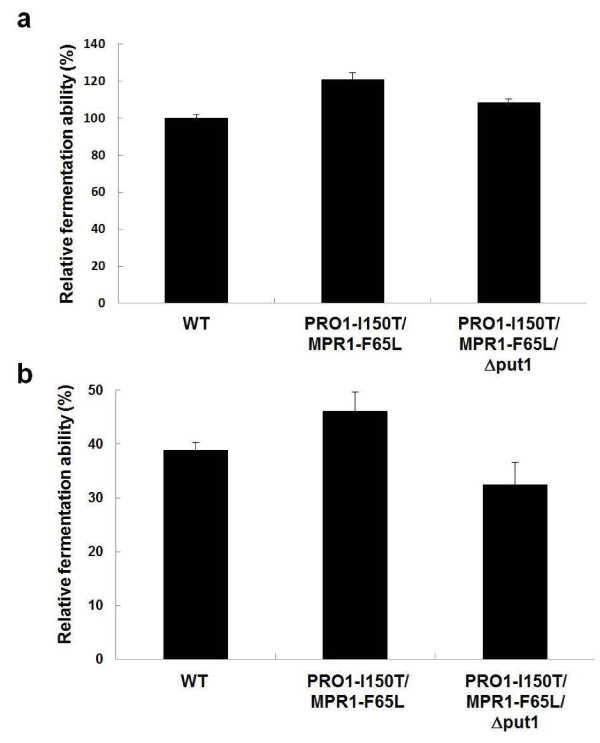
**Fermentation abilities of baker's yeast strains in doughs after baking-associated stress treatments**. (**a**) Fermentation ability after air-drying stress. After exposure to 42°C for 90 min, baker's yeast strains (WT, PRO1-I150T/MPR1-F65L, and PRO1-I150T/MPR1-F65L/Δput1) were mixed with dough and fermented. The remaining CO_2 _gas production after 2 h was measured. The gassing power of WT strain is relatively taken as 100%. The values are the means and standard deviations of results from three independent experiments. Significant difference of PRO1-I150T/MPR1-F65L from WT was confirmed by Student's *t *test (*P *< 0.005). (**b**) Fermentation ability after freeze-thaw stress. The doughs were prefermented for 2 h at 30°C and then frozen at -20°C for 3 weeks. The frozen dough was thawed for 30 min at 30°C, and the remaining CO_2 _gas production after 2 h was measured. The gassing power before freezing of each strain was defined as 100%. The values are the means and standard deviations of results from three independent experiments. Significant difference of PRO1-I150T/MPR1-F65L from WT was confirmed by Student's *t *test (*P *< 0.05).

We assayed the fermentation abilities of baker's yeast strains after air-drying and freeze-thaw stress by measuring CO_2 _gas production in the dough using a Fermograph II (Figure 5). Before stress treatment, there were no significant differences in the gassing power among the three strains (data not shown). Figure 5a shows the relative fermentation ability of baker's yeast strains in bread dough after air-drying stress. An approximately 20% increase in the fermentation ability was observed in PRO1-I150T/MPR1-F65L as compared with WT, probably due to the enhancement of proline and NO synthesis. The fermentation ability in PRO1-I150T/MPR1-F65L/Δput1 with proline accumulation only was slightly but significantly higher than that in WT.

We also assessed the relative fermentation ability of baker's yeast strains in bread dough after freeze-thaw stress (Figure 5b). The remaining gassing power of WT was dramatically decreased to 39% of that before freezing. It is noteworthy that PRO1-I150T/MPR1-F65L showed approximately 20% greater leavening activity than that of WT. On the other hand, the fermentation ability in PRO1-I150T/MPR1-F65L/Δput1 was virtually unchanged from that of WT.

## Discussion

For the baking industry, a yeast strain with tolerance to multiple baking-associated stresses is needed. Many researchers have reported yeast strains that are tolerant only to a single baking-associated stress. For example, antioxidant enzymes, metal ions, and glutathione confer desiccation stress tolerance [24-26] and trehalose, glycerol, and molecular chaperones increase tolerance to freeze stress [27-29]. However, very little is known about multiple baking-associated stress tolerant strain so far [30]. We showed here that the simultaneous expression of Pro1-I150T and Mpr1-F65L in the presence of functional Put1 increases the intracellular NO level in baker's yeast. As a result, increased NO confers high tolerance and fermentation ability on baker's yeast after multiple baking-associated stress treatments. It is noteworthy that these phenotypes were completely abolished in the absence of functional Put1, indicating that Put1- and Mpr1-dependent NO generation is involved in enhanced stress tolerance and fermentation ability during baking-associated stresses. This study is the first to clarify the existence and the importance of a NO-mediated stress tolerant-mechanism in industrial baker's yeast. In addition, we demonstrated the enhancement of the fermentation ability in bread dough after multiple baking-associated stresses.

The PRO1-I150T/MPR1-F65L strain showed a decreased intracellular ROS level and increased an intracellular NO level during air-drying stress (Figure 4). We could not observe any significant differences in intracellular ROS or NO levels after freeze-thaw stress among the three strains we tested. It is difficult to measure intracellular ROS and NO levels during freezing because it is likely that ROS and NO generation occurs in a brief moment. However, based on the result that the PRO1-I150T/MPR1-F65L strain showed higher freeze-thaw stress tolerance and fermentation ability in frozen dough than those of other strains, we believe that Put1- and Mpr1-dependent NO production from proline occurs even under freeze-thaw stress condition as well as air-drying stress. Takahashi *et al. *[31] reported that the deficiency of the transcription activator Mac1, which induces the copper ion transporter genes, caused sensitivity to freeze-thaw stress. Our recent studies suggest that Mac1 is one of the target proteins of NO-mediated *S*-nitrosylation (unpublished observations). *S*-Nitrosylated Mac1 may activate the transcription of the target genes and confer freeze-thaw stress tolerance by maintaining copper ion homeostasis. These data also support our hypothesis that NO is generated during freezing and plays an important role in freeze-thaw stress tolerance.

The reason why the PRO1-I150T/MPR1-F65L/Δput1 strain did not show high leavening activity in frozen dough despite its low intracellular ROS level might be that there was an excess amount of intracellular proline. In this study, cane molasses medium was used for the preparation of yeast cells. For unknown reason(s), a large amount of proline was accumulated in the PRO1-I150T/MPR1-F65L/Δput1 strain after cultivation in cane molasses medium (approximately 18% of dry cell weight). In contrast, when the same strain was cultivated in normal medium such as YPD medium, the proline content was less than 5.0% of dry cell weight. Such an excess of proline might have caused the negative effect on stress tolerance and fermentation ability.

In addition to proline, arginine, and NO, we have clarified that overexpression of the transcription activator Msn2 conferred stress tolerance and fermentation ability on industrial yeasts [32,33]. We also showed the simultaneous accumulation of proline and trehalose in baker's yeast enhanced fermentation ability in the frozen dough compared with the accumulation of only proline or trehalose [34].

On the basis of our findings, it is possible to make breads with greater swelling not only after air-drying but also after freezing, to reduce the freezing period, and to cut the manufacturing cost using the diploid baker's yeast strain PRO1-I150T/MPR1-F65L. The enhancement of proline and NO synthesis by the co-expression of Pro1-I150T and Mpr1-F65L variants described here could be promising for breeding novel baker's yeast strains that are useful for dried yeast production and frozen-dough baking.

## Conclusions

In this work, we clarified the importance of Put1- and Mpr1-mediated NO generation from proline to the baking-associated stress tolerance in industrial baker's yeast. We also demonstrated that baker's yeast that enhances the proline and NO synthetic pathway by expressing the Pro1-I150T and Mpr1-F65L variants showed improved fermentation ability under multiple baking-associated stress conditions. From a biotechnological perspective, the enhancement of proline and NO synthesis could be promising for breeding novel baker's yeast strains.

## Methods

### Yeast strains

In this study, the Japanese diploid baker's yeast strain, derived from 3346 (*MAT*a) and 3347 (*MAT*α), was used as the wild-type strain, WT. To construct the diploid baker's yeast strains that accumulate proline, the haploid strain 3346-ura3 and 3347-ura3 [20] were transformed by integrating linearized pRS406-I150TPRO1 [14] to replace the *PRO1 *gene by the *PRO1-I150T *allele. The *PRO1 *gene encodes γ-glutamyl kinase (GK), and the GK activity is subjected to feedback inhibition by proline [35]. The I150T-mutant GK is much less sensitive to proline-feedback inhibition than the wild-type GK, allowing cells to accumulate proline.

From the resultant proline-accumulating haploid strains (3346 PRO1-I150T and 3347 PRO1-I150T), 3346 PRO1-I150T ura3- and 3347 PRO1-I150T ura3- were obtained by spontaneous mutation from 5-fluoroorotic acid-containing plates. In order to express Mpr1-F65L, the *Sna*BI linearized pRS406-F65L [20] was integrated into the *MPR2 *locus of both 3346 PRO1-I150T ura3- and 3347 PRO1-I150T ura3- by homologous recombination. The Ura^+ ^transformants grown on SD medium were cultured in YPD medium at 30°C for 24 h with shaking, diluted to the same media, and incubated for several days. Then, we obtained strains 3346 PRO1-I150T/MPR1-F65L ura3- and 3347 PRO1-I150T/MPR1-F65L ura3- that have excised the plasmid and lost one of the two copies of the duplicated region by homologous crossover were obtained from 5-fluoroorotic acid-containing plates. In order to confirm whether the residual *MPR *gene on the chromosome was the mutated *MPR1-F65L*, the *MPR *gene region was amplified by genomic PCR and directly sequenced. In order to complement uracil auxotrophy of strains 3346 PRO1-I150T/MPR1-F65L ura3- and 3347 PRO1-I150T/MPR1-F65L ura3-, the linearized pRS406 was integrated into the *URA3 *locus of these strains, and the resultant uracil prototroph strains were named 3346 PRO1-I150T/MPR1-F65L and 3347 PRO1-I150T/MPR1-F65L, respectively. From these two haploid strains, the diploid strain PRO1-I150T/MPR1-F65L was constructed by the method described previously [20].

### Media

Yeast cells were grown in a nutrient rich YPD medium (2% glucose, 2% Bacto peptone [Difco Laboratories, Detroit, MI], and 1% Bacto yeast extract), a synthetic minimal SD medium (2% glucose, 0.67% Bacto yeast nitrogen base without amino acids [Difco Laboratories]), cane molasses medium (5.88% NEO MOLASSEST [EM laboratory, Shizuoka, Japan], 0.193% urea, and 0.046% KH_2_PO_4_), and liquid fermentation medium (5% sucrose, 5% maltose, 0.25% (NH_4_)_2_SO_4_, 0.5% urea, 1.6% KH_2_PO_4_, 0.5% Na_2_HPO_4_-12H_2_O, 0.06% MgSO_4_, 22.5 μg/ml nicotinic acid, 5.0 μg/ml pantothenic acid, 2.5 μg/ml thiamine, 1.25 μg/ml pyridoxine, 1.0 μg/ml riboflavin, and 0.5 μg/ml folic acid). The liquid fermentation medium mimicked prefermentation in dough [36].

### Air-drying stress condition

Yeast cells were grown to the stationary phase in 1 ml of YPD medium at 30°C with reciprocal shaking. Cell cultures (1 ml) were inoculated to 20 ml of cane molasses medium and then cultivated at 30°C for 48 h with rotary shaking at 140 rpm. The harvested cells were washed twice with water, and approximately 1.5 × 10^7 ^cells were collected on sterilized-membrane filter. The filters were subjected to air-drying stress at 42°C for 90 min using a hybridization incubator (HB80, TAITEC, Saitama, Japan).

### Freeze-thaw stress condition

Yeast cells grown on cane molasses medium were washed twice with water, and inoculated to 20 ml of liquid fermentation medium at a final OD_600 _= 1.0. After 4 hours cultivation at 30°C at 140 rpm, cell cultures were divided in aliquots and frozen at -20°C for 2 weeks. The frozen cell suspensions were thawed at 30°C in a water bath for 20 min.

### Cell viability test

Yeast cells grown on cane molasses medium were spotted onto SD plate containing 1 mM hydrogen peroxide (Wako, Osaka, Japan). After air-drying stress or freeze-thaw stress treatment, cell suspensions were serially diluted, spotted onto YPD plate, and then incubated for 1 or 2 days at 30°C.

### Measurement of intracellular proline level after air-drying stress

Yeast cells cultivated in cane molasses medium were harvested, washed twice with water and resuspended in 0.5 ml of distilled water. The suspension was transferred to boiling water and intracellular amino acids were extracted for 20 min. The supernatants were subjected to measurement of proline by an amino acid analyzer (JLC-500, JEOL, Tokyo, Japan). Proline content was expressed as a percentage of dry weight.

### Measurement of intracellular ROS level after air-drying stress

The intracellular ROS level was measured before and after 30 min air-drying stress treatment by the method described previously [19]. The oxidant-sensitive probe 2', 7'-dichlorofluorescin diacetate (Molecular Probes, Eugene, OR) was used for measuring ROS level.

### Measurement of intracellular NO level after air-drying stress

After air-drying stress for 30 min, cells were disrupted with glass beads in a Multi-Beads Shocker (MB601U, Yasui Kikai, Osaka, Japan) for preparation of whole cell extract. The measurement of intracellular NO level was performed using NO_2_/NO_3 _Assay Kit-FX (Dojindo Laboratories, Kumamoto, Japan). The intracellular NO levels were normalized by protein amount.

### Measurement of fermentation ability

For measurement of fermentation ability after air-drying stress, stationary-phase cells grown in cane molasses medium were washed twice with distilled water, and excess water was removed using a porous plate (Nikkato, Osaka, Japan). The filter was placed at 4°C for 1 h, and the cells were air-dried using an air-circulation dryer (Yamato Scientific Co., Ltd., Tokyo, Japan) at 37°C for 90 min and mixed with dough. The formula of dough was 100 g of bread-making flour, 5 g of sucrose, 2 g of NaCl, 4 g of yeast (66% moisture basis), and 70 ml of water. The ingredients were mixed for 3 min with a Swanson type mixer (National Mfg. Co., Ltd., Sterling, IL). The mixed dough was divided into pieces (40 g each) and kept in screw cap bottles. The fermentation ability was assayed by measuring CO_2 _gas production in the dough using a Fermograph II (Atto, Tokyo, Japan) [37].

For measurement of fermentation ability after freeze-thaw stress, stationary-phase cells grown in cane molasses medium were washed twice with distilled water, and used for following dough fermentation. The formula of dough was 100 g of bread-making flour, 5 g of sucrose, 2 g of NaCl, 4 g of yeast (66% moisture basis), and 68 ml of water. The ingredients were mixed for 3 min with a Swanson type mixer. The mixed dough was divided into pieces (40 g each) and placed into screw-cap bottles. After the prefermentation for 2 h at 30°C, the dough was stored at -20°C and kept frozen for 3 weeks. The frozen dough was thawed for 30 min at 30°C, and the fermentation ability was assayed by measuring CO_2 _gas production using a Fermograph II. The freeze tolerance was expressed as the percentage of fermentation ability remaining after freezing relative to the ability before freezing.

## Competing interests

The authors declare that they have no competing interests.

## Authors' contributions

YS, YH, and KH carried out the experimental studies. YS and HT wrote the manuscript. HT conceived the project, co-supervised IO and JS, and checked the data. All authors read and approved the final manuscript.
